# *Nono* deficiency impedes the proliferation and adhesion of H9c2 cardiomyocytes through Pi3k/Akt signaling pathway

**DOI:** 10.1038/s41598-023-32572-x

**Published:** 2023-05-02

**Authors:** Yu-Qing Lei, Zhou-Jie Ye, Ya-Lan Wei, Li-Ping Zhu, Xu-Dong Zhuang, Xin-Rui Wang, Hua Cao

**Affiliations:** 1 Fujian Maternity and Child Health Hospital, College of Clinical Medicine for Obstetrics and Gynecology and Pediatrics, Fujian Medical University, Fuzhou, 350000 China; 2 NHC Key Laboratory of Technical Evaluation of Fertility Regulation for Non-Human Primate (Fujian Maternity and Child Health Hospital), Fuzhou, 350000 China; 3 Department of Cardiac Surgery, Fujian Children’s Hospital (Fujian Branch of Shanghai Children’s Medical Center), College of Clinical Medicine for Obstetrics and Gynecology and Pediatrics, Fujian Medical University, Fuzhou, 350011 China

**Keywords:** Cell biology, Genetics, Congenital heart defects

## Abstract

Congenital heart disease (CHD) is the most common type of birth defect and the main noninfectious cause of death during the neonatal stage. The non-POU domain containing, octamer-binding gene, *NONO*, performs a variety of roles involved in DNA repair, RNA synthesis, transcriptional and post-transcriptional regulation. Currently, hemizygous loss-of-function mutation of *NONO* have been described as the genetic origin of CHD. However, essential effects of *NONO* during cardiac development have not been fully elucidated. In this study, we aim to understand role of *Nono* in cardiomyocytes during development by utilizing the CRISPR/Cas9 gene editing system to deplete *Nono* in the rat cardiomyocytes H9c2. Functional comparison of H9c2 control and knockout cells showed that *Nono* deficiency suppressed cell proliferation and adhesion. Furthermore, *Nono* depletion significantly affected the mitochondrial oxidative phosphorylation (OXPHOS) and glycolysis, resulting in H9c2 overall metabolic deficits. Mechanistically we demonstrated that the *Nono* knockout impeded the cardiomyocyte function by attenuating phosphatidyl inositol 3 kinase-serine/threonine kinase (Pi3k/Akt) signaling via the assay for transposase-accessible chromatin using sequencing in combination with RNA sequencing. From these results we propose a novel molecular mechanism of *Nono* to influence cardiomyocytes differentiation and proliferation during the development of embryonic heart. We conclude that *NONO* may represent an emerging possible biomarkers and targets for the diagnosis and treatment of human cardiac development defects.

## Introduction

Congenital heart disease (CHD), a congenital malformation provoked by fetal cardiac and adjacent large vascular dysplasia, is the leading non-infectious reason of mortality in newborns. At present, it has been a top ranked birth defect, and the worldwide prevalence has increased to 9.410‰^1,2^. Cardiac development is a temporally and spatially restricted process, including differentiation, proliferation, migration and apoptosis of mesoderm-derived cardiac stem progenitor cells^3^. Any imbalance in the complex and precise procedure will induce congenital heart defects^4^. Although the etiology of CHD has both genetic and environmental contributions^5^, the epidemiology of CHD points to genetics contributing to the majority of CHD^6^. Recently, with the development of next-generation sequencing technology, many genes have been identified to be reproducibly associated with CHD^6–8^. It is therefore pivotal to identify the genes that function to regulate the cardiac developmental process.

The non-POU domain containing octamer-binding gene (*NONO*) is located on the X-chromosome and its encoded protein belongs to the Drosophila behavior/human splicing (DBHS) family^9^. The structure of NONO protein determines its molecular scaffold function. As a multifunctional nuclear protein, NONO performs a variety of tasks involved in RNA synthesis, transcriptional activity, post-transcriptional regulation and DNA repair^10^. Emerging clinical evidences^11–17^ showed that the loss-of-function variants of *NONO* could lead to left ventricular non-compaction (LVNC) and CHD, such as atrial septal defect (ASD), ventricular septal defect (VSD), Patent ductus arteriosus (PDA), pulmonary stenosis/pulmonary atresia (PS/PA), Ebstein's anomaly and Hypoplastic left heart syndrome in males. These suggested that *NONO* deficiency may influence cardiac development and lead to impaired cardiac structure and function. Recently, Xu et al. found that myocardial fibroblast hyperplasia and excessive collagen secretion may be the cause of cardiac defects in *Nono* KO mice^18^. However, the effects of *NONO* on cardiac development have not been fully elucidated. Cellular landscape of the human heart shows cardiomyocytes make up the largest proportion of the heart^19^. Anomalies in cardiomyocyte proliferation and disruption of myocardial wall morphogenesis can drive various forms of CHD and non-compacting cardiomyopathy^20,21^.

In this study, we explore the effect of *Nono* deficiency on Rat H9c2 embryonic cardiomyocytes cultured in vitro by comparing wild type (WT) cardiomyocytes and Nono gene knockout (Nono-KO) cardiomyocytes. We investigated the differences in growth, proliferation, adhesion, and energy metabolism of the two types of cells and further explored the mechanism by the assay for transposase-accessible chromatin using sequencing (ATAC-seq) coupled with the RNA sequencing (RNA-seq). Our research may initially provide a new direction for expanding and improving prenatal CHD diagnosis and treatment strategies.

## Results

### *Nono* knockout experiments in cultured rat cardiomyocytes H9c2 by CRISPR-Cas9

**Figure 1:**
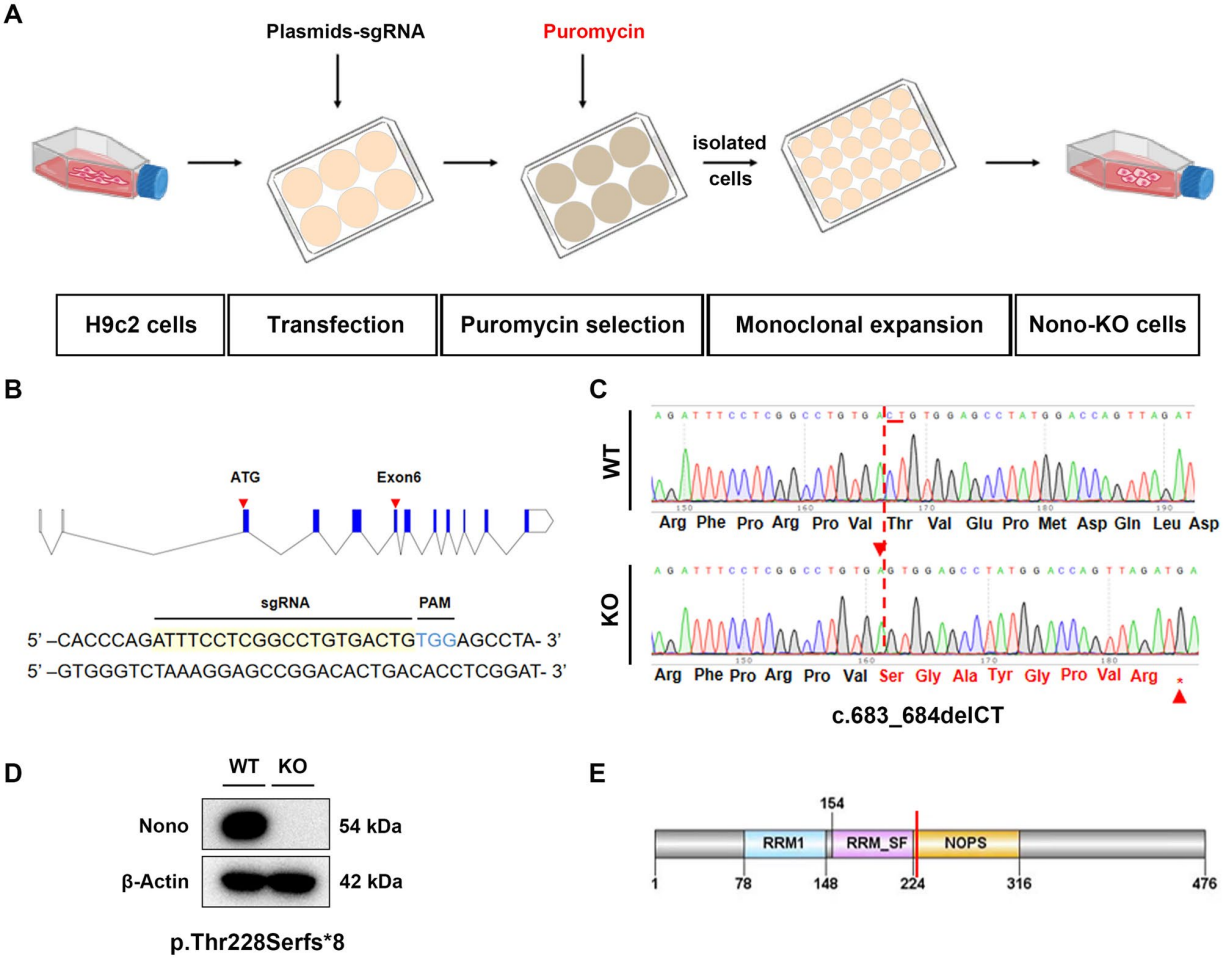
Construction and identification of *Nono* knockout H9c2 Cell Line. (**A**) Schematic diagram of Nono-KO cell line construction by CRISPR/Cas9 gene editing system. (**B**) A sgRNA sequence located at exon 6 was designed for rat Nono gene to delete nucleotides in the target sequence. (**C**) Alignment of DNA sequencing traces of Nono-KO to WT H9c2 cells. The red dotted line indicates nucleotide deletions in Nono-KO cell lines. The red arrows indicate the deleted sites. (**D**) Western blotting analyses of selected WT and Nono-KO H9c2 cell clones with Nono and β-actin (loading control) antibodies. (**E**) Pattern of Nono protein. The red line indicates the translation termination site. **sgRNA**, single guide RNA. **PAM**, protospacer adjacent motif. **c.683_684delCT**, Deletion of bases C and T at 683 to 684 of rat Nono gene encoding region. **p.** Thr228Serfs*8, The missense mutation of threonine at 228 of Nono protein to serine leads to the termination of protein translation. The original bands of Western blotting from Fig. 1D were shown in the Supplementary Fig. 3.

We generated Nono-KO cell line with a homozygous knockout of *Nono* by targeting exon 6 of rat Nono gene with the CRISPR/Cas9 gene editing system (Fig. 1A,B). To validate successful *Nono* knockout, we performed target site sanger sequencing and western blotting analysis (Fig. 1C,D). Compared with WT, two bases (C and T) were deleted at 683 and 684 of Nono gene in coding-region sequences (Fig. 1C), which resulted in missense mutation (threonine mutation to serine) at 228 and nonsense mutation at 236 of Nono protein (Fig. 1D), and ultimately caused early termination of Nono protein translation (Fig. 1E). These results indicate that we have successfully constructed a stable Nono-KO H9c2 cell line.

### *Nono* deficiency inhibits cell adhesion, cell proliferation and cell cycle progression in H9c2 cell

**Figure 2:**
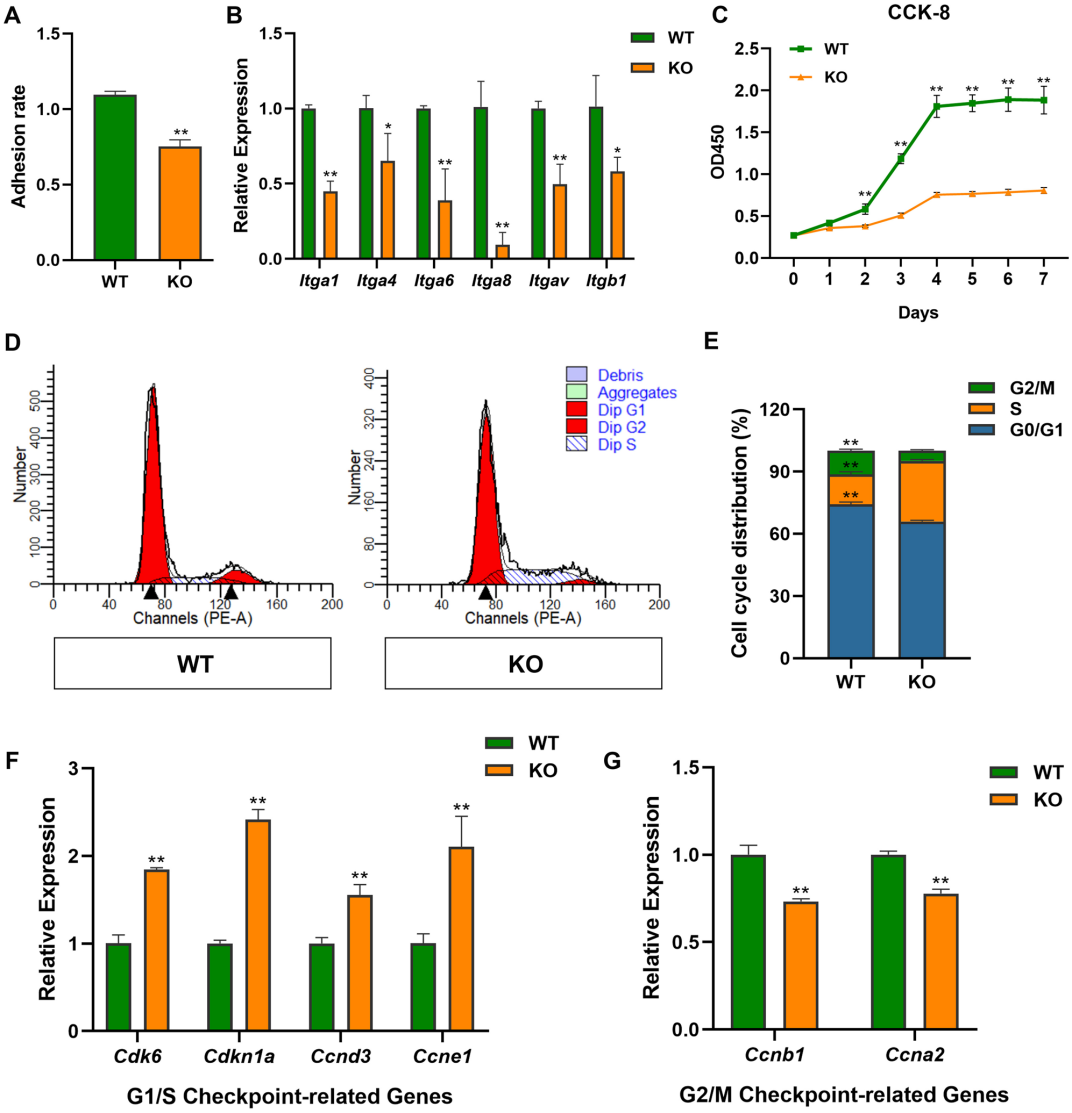
The effects of *Nono* on H9c2 cell proliferation, adhesion and cell cycle progression. (**A**) Rate of adhesion between WT cells and Nono-KO H9c2 cells. (**B**) Expression of cell adhesion-related Itg-family genes detected by qPCR (relative to GAPDH). (**C**) CCK-8 assay showing that *Nono* knockout inhibits H9c2 cardiomyocytes viability, and proliferation of *Nono* deficient cardiomyocytes decreased significantly. (**D**) Flow cytometry analysis of DNA content shows G0/G1, S and G2/M phase distribution of H9c2 WT and KO cells. *Nono* knockout causes S-phase arrest. (**E**) Histogram of the percentages of G0/G1, S and G2/M cells. (**F**) Expression of G1/S checkpoint-related genes detected by qPCR (relative to GAPDH). (**G**) Expression of G2/M checkpoint-related genes detected by qPCR (relative to GAPDH). In all panels, the data are representative of three independent experiments. The data are presented as the mean ± SD. Statistical significance is shown as *, *P* < 0.05; **, *P* < 0.01 vs. control.

To explore the adhesion rate of H9c2 cardiomyocytes after Nono gene knockout, we investigated the cellular adhesion rate of WT and KO H9c2 cells. The result shown that *Nono* deficiency inhibits cell adhesion (Fig. 2A). The mRNA expressions of cell adhesion factor integrin family genes are down-regulated (Fig. 2B). Which indicated that the connection between cardiomyocytes and their external environment is impaired.

To investigate whether *Nono* plays a pivotal role in the development of H9c2 cardiomyocytes, we used CCK-8 to detect cell proliferation in WT and Nono-KO H9c2 cells. After 48-h incubation, KO group significantly decreased the growth rates compared with WT group in H9c2 cells (Fig. 2C). Meanwhile, flow cytometry analysis showed that *Nono* knockout was associated with the G0/G1 population decreasing by 9.73% compared to the control cells (Nono-KO cells, 65.71% vs WT cells, 75.44%) and G2/M population declining by 6.76% (Nono-KO cells, 4.32% vs WT cells, 11.08%). However, *Nono* depletion led to increased fraction of S-phase cells (Nono-KO cells, 29.97% vs WT, 13.48%) (Fig. 2D,E). Subsequently, we quantified the mRNA expression of G1/S and G2/M transition associated genes in two groups. qPCR results showed that the expression of checkpoint-related genes in G1/S phase (Cdk6, Cdkn1a, Ccnd3 and Ccne1) was significantly up-regulated (Fig. 2F), while the expression of checkpoint-related genes in G2/M phase (Ccnb1 and Ccna2) was significantly down-regulated (Fig. 2G). Collectively, these results suggest that *Nono* deficiency inhibits cell proliferation and arrests cells in the S phase.

### *Nono* deletion inhibits energetic metabolism by suppressing mitochondrial OXPHOS and glycolysis in H9c2 cardiomyocytes

**Figure 3:**
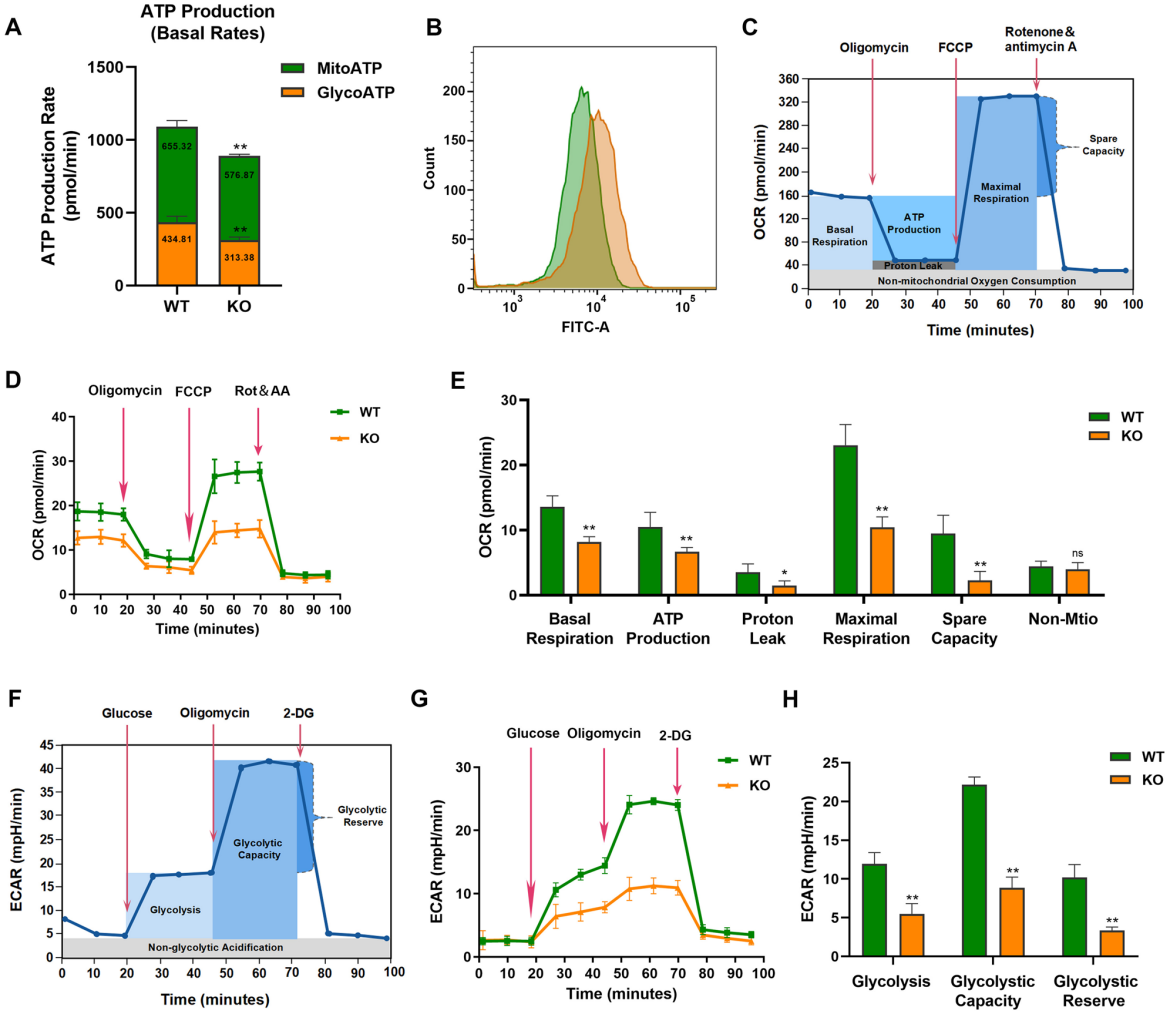
Nono-KO inhibits mitochondrial function and glycolytic capacity in H9c2 cells. (**A**) The ATP production rate in WT and Nono-KO cells was measured using the real-time ATP Rate Assay. Both mitochondrial and glycolytic ATP production were determined from three independent experiments. (**B**) Identification of cellular reactive oxygen species (ROS) between WT and KO group using DCFH-DA staining and flow cytometry. (**C**) Cell mitochondrial stress test profile plot. (**D**) Mitochondrial respiration in WT and Nono-KO cells was monitored using the Mitochondrial Stress Test. Trace shows representative data from one of three experiments. (**E**) Quantification of the mitochondrial respiration data for basal respiration, ATP production, proton leak, maximal respiration, spare respiratory capacity and non-mitochondrial oxygen consumption, which obtained from three independent experiments. (**F**) Cell glycolytic stress test profile plot. (**G**) The glycolysis process in WT and Nono-KO cells was monitored using the Glycolysis Stress Test. Trace shows representative data from one of three experiments. (**H**) Quantification of the glycolytic stress data for glycolysis, glycolytic capacity and glycolytic reserve from three independent experiments. The data are presented as the mean ± SD. Statistical significance is shown as ns, no significance; *, *P* < 0.05; **, *P* < 0.01 vs. control. **MitoATP**, ATP produced by mitochondria. **GlycoATP**, ATP produced by glycolysis.

The energy synthesis of cardiomyocytes can maintain the stability of the metabolism of basic substances in the heart, further maintain the function of the heart and meet the metabolic needs of various organs of the body. The deletion of *Nono* can decrease total cellular ATP content by influence both OXPHOS and glycolysis (Fig. 3A).

To explore the effect of *Nono* on oxidative stress production in H9c2 cardiomyocytes, the ROS production between WT and Nono-KO cells was measured. For those *Nono*-silencing cells, the ROS production were increased compared with WT one (Fig. 3B). Subsequently, we further researched the role of *Nono* in mitochondrial oxidative respiration and glycolysis of rat H9c2 cardiomyocytes. The value of OCR and ECAR in two groups were measured by Seahorse XFp analyzer. Four drugs affecting mitochondrial function were injected during the mitochondrial stress test. Each data point represents the average of three measurements at each time point (Fig. 3C). The results indicated that compared with WT group, mitochondrial OXPHOS in Nono-KO group was reduced by OCR measurement (Fig. 3D). Meanwhile, we observed that *Nono* deficiency could significantly reduce the basal respiration (*P* < 0.01), ATP production (*P* < 0.01), proton leak (*P* < 0.05), maximal respiration (*P* < 0.01) and spare capacity respiration (*P* < 0.01) in H9c2 cells compared with WT group. However, there was no significant difference in the non- mitochondrial oxygen consumption between the two groups (Fig. 3E). The proton leak was calculated with OCR values under the coupling and uncoupling states. We found that Nono gene knockout could reduce the proton leak in both coupling and uncoupling states (Supplementary Fig. 1). Proton leak is the result of the opening of mitochondrial permeability transition pore (MPTP) in mitochondrial inner membrane^22^.

The glycolysis of H9c2 cells with knockout of *Nono* was also examined by XFp Seahorse analysis (Fig. 3F). The data showed that deletion of Nono gene down-regulates the glycolysis of H9c2 cells (Fig. 3G). Likewise, the glycolytic capacity (*P* < 0.01) and glycolytic reserve (*P* < 0.01) decreased significantly (Fig. 3H).

Consistently, gene set enrichment analysis (GSEA) showed that knocking out the Nono led to suppressed expression of a glycolysis gene signature and a oxidative phosphorylation gene signature (Supplementary Fig. 2). Analysis of cellular energy metabolism showed that Nono deficiency suppressed OXPHOS and glycolysis, resulting in H9c2 cardiomyocytes overall metabolic deficits.

### The differentially expressed genes (DEGs) between H9c2 WT and Nono-KO cells

**Figure 4:**
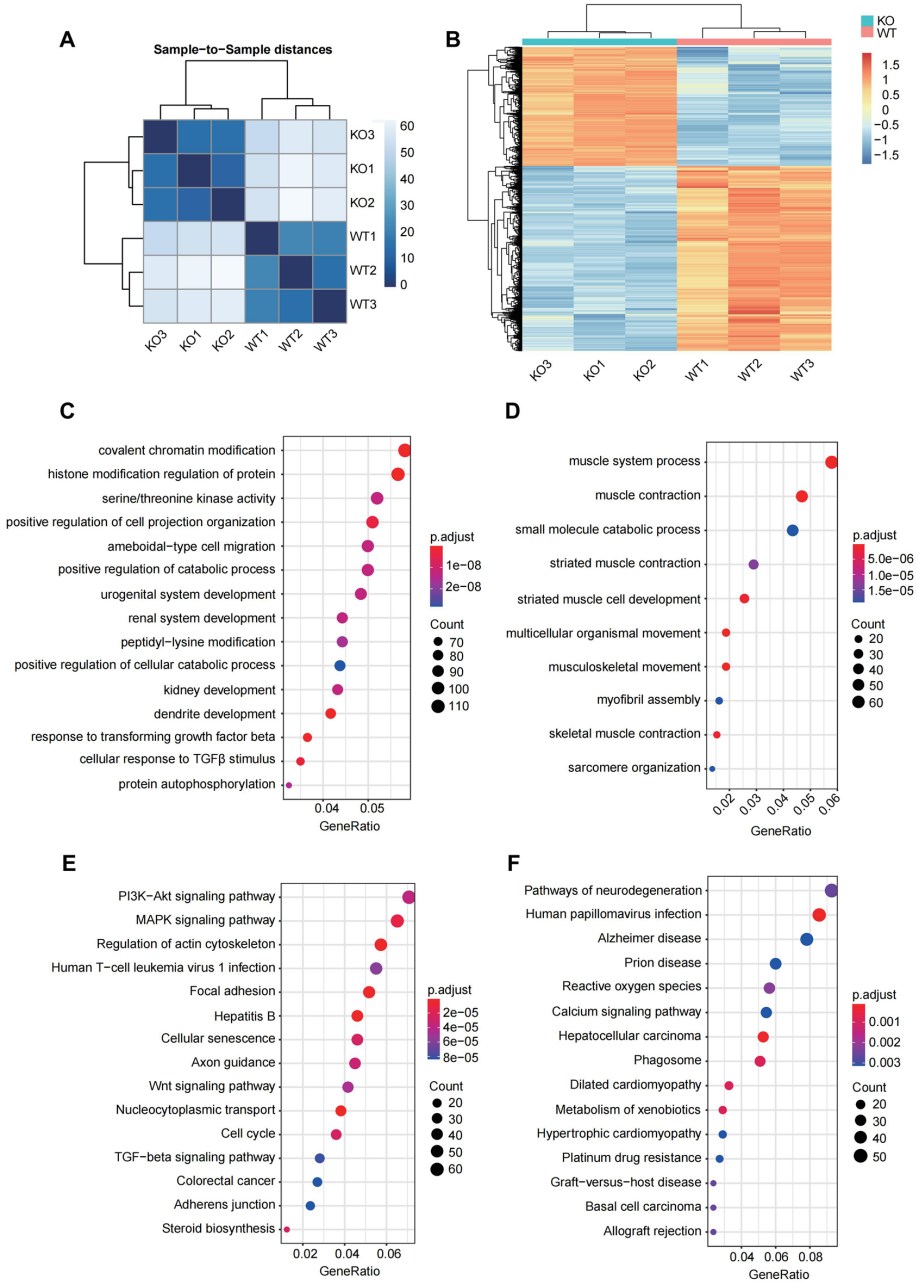
The DEGs between H9c2 WT and Nono-KO cells. (**A**) Sample-to-sample distances generated with DeSeq2 software packages showing the Euclidean distances between the samples. (**B**) Heatmap of differentially expressed genes (defined as |log fold-change (logFC)|> 1, with a FDR < 0.01) in *Nono* knockout H9c2 cells relative to WT cells. (**C**,**D**) GO analysis of the identified down-regulated (left) or up-regulated (right) DEGs. (**E**,**F**) Pathway analysis using KEGG was performed on the down-regulated (left) or up-regulated (right) DEGs.

The transcriptome between H9c2 WT and Nono-KO cells was compared and analyzed of, and a strong positive correlation was detected between repetitions within each cell types (Fig. 4A). Compared with H9c2 WT cells, 1374 up-regulated DEGs and 2131 down-regulated DEGs were identified in Nono-KO cells (Supplementary Dataset File 1). The heatmap showed significant DEGs (Fig. 4B). The Gene Ontology (GO) analysis revealed that down-regulated DEGs were associated with chromatin modification and histone modification regulation, up-regulated DEGs were associated with muscle system process and muscle contraction (Fig. 4C,D, Supplementary Dataset File 2, 3). Additionally, KEGG analysis demonstrated that the down-regulated DEGs were significantly enriched in the Pi3k-Akt and MAPK signaling pathway, up-regulated DEGs were enriched in the human disease pathway (Fig. 4E,F, Supplementary Dataset File 4, 5).

### The Differentially accessible regions (DARs) between H9c2 WT and Nono-KO cells

**Figure 5:**
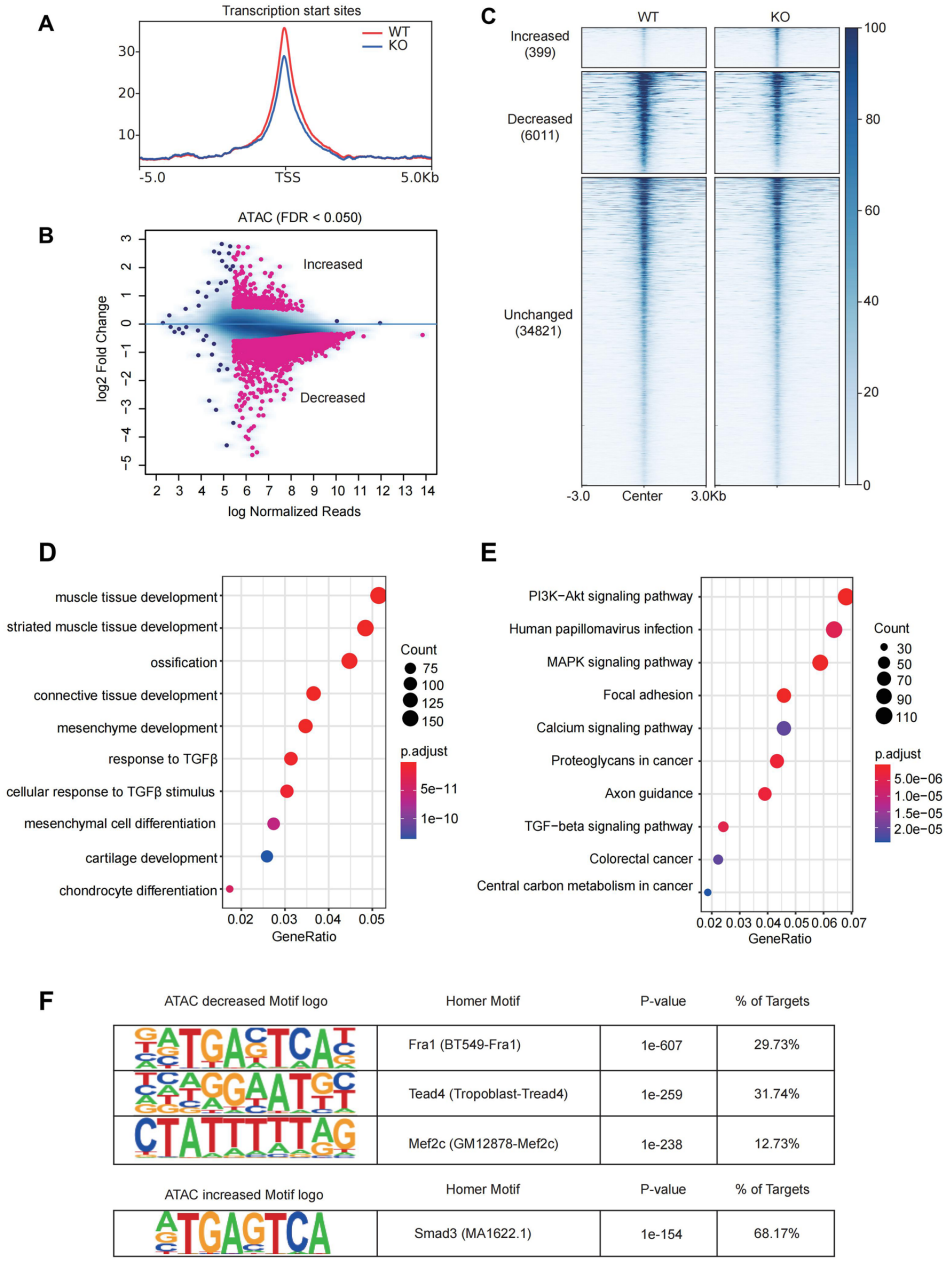
The DARs between H9c2 WT and Nono-KO cells. (**A**) Signal intensity plot representing changes in ATAC-seq signal at TSS regions following knockout of *Nono* in H9c2 cells. The enriched regions were extended ± 5 kb from their midpoint. (**B**) Scatter plot showing DARs between H9c2 WT and Nono-KO cells. (**C**) Heatmap analysis showing read density of ATAC-seq peaks in H9c2 WT and Nono-KO cells. (**D**) Biological process analysis of genes associated with decreased DARs. Top 10 significantly enriched GO terms are shown. (**E**) KEGG pathway analysis of genes associated with decreased DARs. Top 10 significantly enriched GO terms are shown. (**F**) Motif analysis of genes associated with decreased (top) or increased (bottom) DARs.

To measure the impact of genome-wide accessible regions of *Nono*-regulated chromatin architecture, we investigated chromatin accessibility by ATAC-seq. The TSS and its surrounding region (± 5000 bp) of all annotated genes are relatively less accessible in Nono-KO cells (Fig. 5A). Compared with WT, the majority of peaks (34,821 peaks, ~ 84%) were unchanged, while we identified 399 increased DARs and 6011 decreased DARs in Nono-KO cells (Fig. 5B,C, Supplementary Dataset File 6). The GO analysis of the genes near the peak reduction site indicated that genes in DARs were enriched for pathways related to muscle tissue development and muscle cell proliferation (Fig. 5D, Supplementary Dataset File 7). The KEGG enrichment analysis revealed that those genes were involved in Pi3k-Akt signaling pathway and focal adhesion (Fig. 5E, Supplementary Dataset File 8). To dissect the upstream regulators of differentially expressed genes that are associated with differential chromatin accessibility, HOMER motif analysis was conducted. Results for DARs revealed that Fra1 motifs was strongly enriched in H9c2, along with Tead4 and Mef2c (Fig. 5F). On the other hand, increased DARs was preferentially found at genes involved in Smad3.

### *Nono* Knockout disrupts Pi3k-Akt network

**Figure 6:**
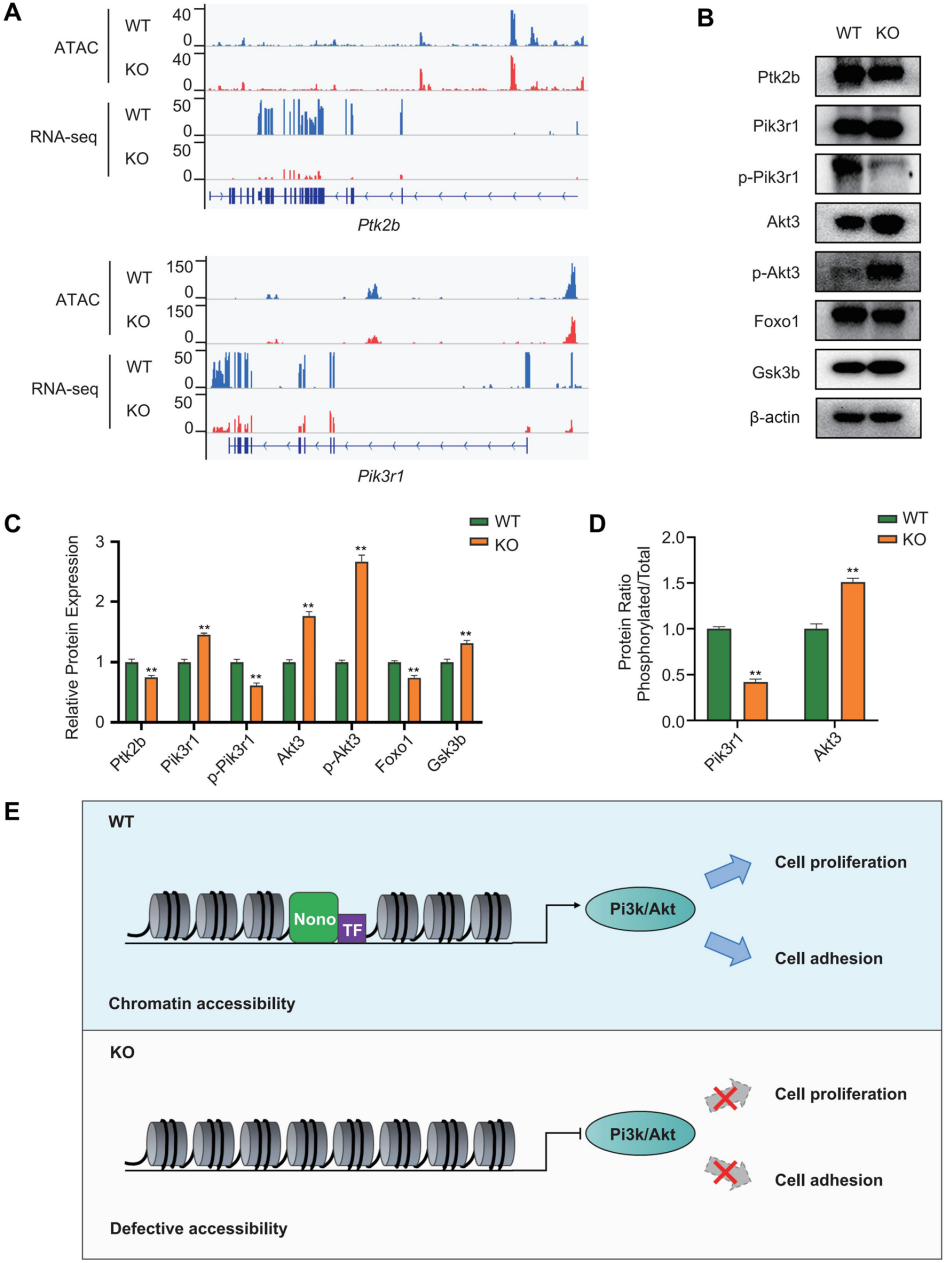
Signaling mechanisms involved in Nono deficient H9c2 cardiomyocytes. (**A**) Genome browser tracks of RNA-seq data and ATAC-seq data at the *Ptk2b* and *Pik3r1* loci following knockout of *Nono* in H9c2 cells. (**B**) Western blotting was used to detect the expression levels of representative molecules in the Pi3k-Akt signaling pathway in H9c2 WT and Nono-KO cell lines. (**C**) Western blotting quantitative analysis of the expression level. (**D**) Western blotting quantification of the phosphorylation rate of *Pik3r1* and *Akt3.* (**E**) Schematic illustration of Nono gene roles in rat cardiomyocytes H9c2. Nono gene knockout led defective chromatin accessibility, and further impeded the proliferation and adhesion in H9c2 cells through Pi3k-Akt signaling pathway. The original bands of Western blotting from Fig. 6B were performed in the Supplementary Fig. 4.

Furthermore, we integrated our ATAC-seq data with and RNA-seq data and examined the impact of the *Nono* knockout on network of Pi3k-Akt. As shown by the genomic browser tracks (Fig. 6A), chromatin openness and gene expression were markedly decreased at the Ptk2b and Pik3r1 loci. We further detected the expression of representative proteins in the Pi3k-Akt signaling pathway in H9c2 wild type cell lines and Nono gene knockout cell lines. The results demonstrated downregulation of Ptk2b, p-Pik3r1/Pik3r1 ratio, and Foxo1 in Nono-KO cell lines compared to those in normal H9c2 cells. On the other hand, the expression of p-Akt3/Akt3 ratio and Gsk3b was enhanced in Nono-KO H9c2 cardiomyocytes (Fig. 6B). In addition, Nono knockout reduced the protein levels of Ptk2b, p-Pi3kr1, Foxo1 (*P* < 0.01), and increased the protein levels of Pik3r1, Akt3, p-Akt3 and Gsk3b (*P* < 0.01) (Fig. 6C,D). These data demonstrated that the overall activity of the Pi3k/Akt signaling pathway in the Nono-KO cells was affected compared to the wild type H9c2 cardiomyocytes. Taken together, our results reveal the molecular mechanisms by which Nono regulates the function of H9c2 embryonic cardiomyocytes (Fig. 6E).

## Discussion

Disturbances in heart development contribute to variety multifarious malformation. CHD is one of the most common human birth defects in the world, which can be caused by environmental exposure coupled with genetic susceptibility. Emerging evidences have illustrated that environmental factors induce only 2%–5% of CHD and that the majority of patients are associated with heredity or genetic defects^23^. Genetic etiology researches are difficult to carry out as the existence of unknown CHD-related genes, the genetic diversity of human subjects and genetic heterogeneity of CHD^24^. Therefore, it is necessary to further explore the potential CHD-related genes and their functions in regulating embryonic cardiac development.

With roles in almost every step of gene regulation^10^, *Nono* has diverse molecular functions, and it is gradually founded the clinically relevant in the contexts of cancer^25^, Innate immunity^26^, neurodevelopment^27^. Recently, it is reported worldwide that fifteen cases with cardiac phenotypes of LVNC and CHD have been found *NONO* loss-of-function variants^11–17^. To this date, there are no reports on the cardiac development of *NONO*. Therefore, we surmised that multi-functional factor *NONO* might play a role in the occurrence of CHD. Cardiac development through differentiation of multipotent cardiac progenitor cells into cardiomyocytes and local proliferation of cardiomyocytes^28^. So cardiomyocytes play a pivotal role in development and composition of heart.

In the present study, we examined the role of *Nono* in cardiomyocytes during development using a CRISPR-Cas9 gene editing approach in vitro. Results showed that Nono-KO H9c2 cell line have lessened proliferation and adhesion capacity, impaired mitochondrial function and energy metabolism. It is, for the first time, revealed an adverse impact of Nono gene deficiency on cardiomyocytes development in vitro. The findings suggested that deleted Nono gene could cause defective chromatin accessibility, and further impede embryonic cardiomyocyte function via Pi3k-Akt signaling pathway. This study provides a preliminary mechanism exploration between cardiomyocytes disfunction and Nono gene loss-of-function, and also extends our knowledge and prompts us to rethink more regarding Nono gene roles in embryonic heart development in vivo.

Cardiomyocytes proliferation is essential in heart development, including shaping cardiac structures^29^. We examined the effect of *Nono* expression on the growth of H9c2 cells first. Abnormal cell cycle related to effectively decreased proliferation of cardiomyocytes were observed in H9c2 cell lines with *Nono* deficiency. Flow cytometry experiment was used subsequently to identify the cell cycle stage at which *Nono* has a role. Our results demonstrated more than twofold increase of cells in S phase of the cell cycle when *Nono* was absent. Meanwhile, we observed up-regulated expression of Cdk6, Cdkn1a, Ccnd3, Ccne1 (checkpoint-related genes in G1/S phase) and down-regulated expression of Ccnb1, Ccna2 (checkpoint-related genes in G2/M phase). Consistently, Xu et al.^18^ founded that the primary ventricular fibroblasts of *Nono* deficient adult mice increased significantly in S-phase. In addition, they demonstrated that Nono gene knockout resulted in impaired cardiac function and fibrosis in mice^30^.

The integrins are transmembrane proteins encoded by α and β subtypes that form functional heterodimers, bind components of the extracellular matrix (ECM), and transmit intracellular signals upon ligand binding^21^. Cardiac fibroblasts produced ECM components and acted as a scaffold^31^. Cross talk between the myofibroblast and the cardiomyocyte have an potential impact on the structure and function of the myocardium^32^. Ieda et al.^33^ believed that the specific signals of embryonic cardiac fibroblasts synergistically promoted cardiomyocyte proliferation in a paracrine manner. But this proliferative response required myocardium β1-integrin (Itgβ1), mouse ventricular myocyte-specific deleted Itgβ1 led to decreased myocardial proliferation and impaired ventricular compactness. This is consistent with the impaired cell adhesion and significantly decreased expression of integrin family related proteins found in *Nono* knockout cardiomyocytes.

Considering that the heart has a voracious requirement for energy^34^. Besides, Numerous studies have shown that *NONO* regulates energy metabolism in cancer cells^35,36^. We detected the total cellular ATP content, mitochondrial and glycolysis stress tests utilizing the Seahorse XF technology. Data showed the total ATP, mitochondrial and glycolytic ATP production were decreased. Moreover, the mitochondrial OXPHOS and glycolysis were reduced in Nono-KO H9c2 cells. Gene‑set enrichment analysis of RNA-seq analysis showing enrichment of the oxidative phosphorylation genes and glycolysis genes. ROS is a physiological byproduct of electron transport in the respiratory chain on the inner mitochondrial membrane, and its generation is increased or uncontrolled if the respiratory chain electron transport on the inner mitochondrial membrane is compromised^37,38^. We further inspected the ROS level in cardiomyocytes and found that increased ROS in *Nono* deficiency H9c2 cells. Taken together, we believe that *Nono* knockout impaired mitochondrial function of cardiomyocytes. Researches have confirmed that changes in metabolic substrates affect cardiomyocyte function (such as Cardiomyocyte differentiation and proliferation)^39,40^. Therefore, we reasonably believe that there is a correlation between the damage of energy metabolism and cell function of cardiomyocytes with Nono gene depletion.

Simultaneously, we integrated chromatin accessibility and transcription analysis in H9c2 WT cells and Nono-KO cells through ATAC-seq combine with RNA-seq, which are mutually authenticating. Based on the analysis of DEGs and their function annotation, we found that related genes participating in the response to TGFβ, the cellular response to TGFβ stimulus, and the cardiac muscle tissue growth process were down-regulated. It is been proved that TGFβ signaling regulates cardiomyocyte proliferation and differentiation via targeting to the important genes of early heart development^41^. In contrast, the mRNA expression levels of covalent chromatin modification and histone modification regulation related genes were up-regulated. We further integrated chromatin accessibility and transcription analysis data in the two group cells. We identified the downstream pathways and genes for *Nono* that may regulate the proliferation and adhesion of H9c2 cardiomyocytes. We found for the first time that depletion of *Nono* in H9c2 embryonic^42^ cardiomyocytes significantly impeded chromatin accessibility, which in turn affected the Pi3k-Akt signaling pathway. Subsequently, we confirmed the effect of *Nono* on this signaling pathway by western blotting. This indicated the underlying mechanisms for *NONO* deficiency to inhibit cell cycle and cell adhesion. It has been proved that the Pi3k-Akt pathway and FOXO transcription factors prompted cardiomyocyte proliferation^43,44^, and associated with cellular oxidative stress gene expression^45^. The upstream regulator of Foxo1, AKT, inhibits Foxo1 activity by phosphorylating Foxo1^46,47^. Moreover, motif analysis of several genes associated with decreased DARs (Fra1, Tead4 and Mef2c). The transcription factor Mef2c determines myocyte fate and development^21^. TEAD transcription factors are required for normal primary myoblast differentiation in vitro^42^. Those are consistent with our findings.

There were, however, two limitations to this study. On the one hand, we only conducted preliminary in vitro experiment, and the role of *Nono* in cardiac development in vivo needs to be further explored. On the other hand, the impaired mitochondrial function mechanisms put forward in this study need more evidence to confirm, and we would make efforts to validate the findings in the near future study in vivo.

## Methods

### Cell culture and cell transfection

Rat cardiomyocytes H9c2 were purchased from American Type Culture Collection (ATCC, cat. no. CRL-1446), cultured in DMEM (Gibco, cat. no. C11995500BT) supplemented with 10% fetal bovine serum (Hyclone, cat. no. SV30208.02) and maintained in a humidified incubator (Thermo Scientific, cat. no. 3131) with 5% CO2 atmosphere at 37 ℃. The medium was changed every 2–3 days. Experiments were carried out with cells in logarithmic growth phase.

The day before transfection, cells (1.0 × 10^6^ cells/well) were seeded in six-well plates and cultured overnight until almost 70–80% cell confluence. The pSpCas9(BB)-2A-Puro (PX459) plasmid vector (available from our lab) was constructed in the sgRNA (5’- caccgATTTCCTCGGCCTGTGACTG), and the pEGFP-N1 plasmid vector (available from our lab) was used as control plasmid. 2 µg plasmid was diluted in 80 µL Opti-MEM (Sigma, cat. no. 408727), 6 µL PEI (Polysciences, cat. no. 23966–1) was diluted in 80 µL Opti-MEM, the two were mixed and incubated at room temperature for 10 min. PEI/plasmid mixture was then added to single well of 6-well plate. After 12 h, green fluorescence was examined under a fluorescence microscope (Nikon Eclipse TSZR) with 10 ×/0.30 objective. Then, Puromycin (Yeasen Biotechnology, cat. no. 60210ES60) was used to screen and achieve the stable cell lines.

### Cell proliferation assay

H9c2 cells proliferation was detected using the Cell Counting Kit-8 (CCK-8) cell proliferation kit (Dojindo, cat. no. CK04) according to the manufacturer’s instructions. H9c2 cells were seeded in 24-well plates. After addition of CCK-8 solution in each well, cells were then cultured for another 2 h in the incubator. The absorbance of 450 nm was detected through microplate spectrophotometer (Agilent, BioTek Epoch). All experiments were performed in triplicate, and three independent repeat experiments were performed.

### Flow cytometry analysis of cell cycle and reactive oxygen species (ROS)

H9c2 cardiomyocytes were harvested by 0.25% trypsin EDTA (Gibco, cat. no. 2323251), rinsed with pre-cooling phosphate-buffered saline (PBS) and fixed with 70% ethanol for 12 h at 4 °C. After washing with PBS for 15 min at room temperature, the cells were treated with RNase A at 37 °C for 30 min and incubated with propidium (PI) staining solution (Elabsciences, cat. no. E-CK-A351) at 4 °C for 30 min.

The ROS assay (Beyotime, cat. no. S0033S) was used to evaluate oxidative stress levels after *Nono* knockout. Cells were fixed with 4% Paraformaldehyde (Solarbio, cat. no. P1110) overnight and rinsed with pre-cooling PBS. Each sample was incubated with 10 μM DCFH-DA (diluted with serum-free DMEM) at 37 °C for 30 min. The positive control group was treated with 10 μM Rosup in advance according to the manufacturer's instruction.

Flow cytometer (BD LSRFortessa™ Cell Analyzer, Cat. no. 647800) was used to measure cell cycle and ROS parameters in H9c2 cells suspensions with the FL1 channel for green fluorescence and the FL3 channel for red fluorescence at excitation wavelength 488 nm.

### Cell-adhesion assay

The Cell Adhesion Detection kit (BestBio, cat. no. BB-48120) was employed to analyze the adhesion ability of H9c2 cells. According to the manufacturer's protocol, the coating buffer (100 µL) was transferred to a 96-well plate and incubated overnight at 4 °C. After removing coating buffer, the plate was rinsed three times with the detergent solution. Cells in the two groups were plated in 96-well plate at the density of 5 × 10^4^ cells per well in triplicates for each group. After 2 h of incubation at 37 °C, the medium was changed and the staining solution B (10 µL) was added to the wells. The OD was detected at 450 nm after 1 h using a microplate spectrophotometer (Agilent, BioTek Epoch). The formula used for calculating the cell adhesion rate was: cell adhesion rate (%) = (OD of knockout cells − OD of the blank) / (OD of the control cells − OD of the blank) × 100.

### XF real-time ATP rate assay

The Real-time ATP Rate assay Kit (Agilent, cat. no. 103592–100) was used to measure the total ATP production. Cells were exposed to these compounds as follows: oligomycin (1.5 μM) and the rotenone/antimycin A mixture (0.5 μM), which can report multiple parameters, including glycolytic ATP, mitochondrial respiratory ATP rate, and total ATP production rates. Data were analyzed using XF Wave Software (Seahorse Bioscience, Agilent, US). All data were normalized to protein content.

### XF cell mitochondrial stress test

Mitochondrial stress was measured according to instructions by the Seahorse XFp Cell Mitochondrial Stress Test Kit (Agilent, cat. no. 103015-100). Briefly, cells were metabolically perturbed by the addition of three compounds in succession, and OCR (Oxygen consumption rate) was measured prior to and after addition of each compound as follows: oligomycin (1.5 μM), carbonyl cyanide-4-(trifluoromethoxy) phenylhydrazone (FCCP) (2 μM), and rotenone/antimycin A mixture (0.5 μM). Data were analyzed using XF Wave software (Seahorse Bioscience, Agilent, US).

### XF glycolysis stress test

According to the instruction of the Seahorse XFp Glycolysis Stress Test Kit (Agilent, cat. no. 103020–100). Cells were exposed to three compounds, with measurements after each addition as follows: glucose (10 mM), oligomycin (1 μM), and 2-deoxy-D-glucose (2-DG, 50 mM). Data were analyzed with XF Wave software.

### RNA extraction, reverse transcription, and real-time quantitative PCR (qPCR)

Total RNA was extracted from H9c2 cells using QIAwave RNA Mini Kit (Qiagen, cat. no. 74536) following the manufacturer's instructions. RNA concentration was measured by NanoDrop™ microspectrophotometer (Thermo Fisher Scientific, cat. no. ND-ONE-W) at 260 nm and 280 nm. For the detection of mRNA expression level, PrimeScript™ RT reagent Kit with gDNA Eraser (Perfect Real Time) (Takara, cat. no. RR047A) was used to form cDNA. SYBR® Green Realtime PCR Master Mix (TOYOBO, cat. no. QPK-201) was applied for qPCR. All the qPCR was conducted on the QuantStudio™ 5 Real-Time PCR System (Thermo Fisher Scientific, cat. no. A34322). The expression of housekeeping gene GAPDH was utilized as a reference to normalize the amount of transcript. The 2^−ΔΔCt^ method^48^ was used to calculated the relative expression levels. All the primers used in this part were listed in additional information (Supplementary Table 1).

### Western blotting

Protein was elicited from H9c2 cells through employing RIPA lysis and extraction buffer (Thermo Fisher Scientific, cat. no. 89900), which encompassed the Protease Inhibitor Cocktail (Cell Signaling Technology, cat. no. 5871). Halt™ Phosphatase Inhibitor Cocktail (Thermo Fisher Scientific, cat. no. 78420) was used to preserve the phosphorylation state of proteins. The concentration of protein was assessed using Pierce™ BCA Protein Assay Kit (Thermo Fisher Scientific, cat. no. 23225). Subsequently, samples were subjected to 10% SDS-PAGE gels, transferred onto Immun-Blot PVDF membranes (Bio-Rad, cat. no. 1620177), blocked with 5% non-fat milk for 2 h at room temperature, and separately immunoblotted with several antibodies such as Ptk2b (Abcam, cat. no. ab32571), Pik3r1 (Abcam, cat. no. ab191606), Akt3 (Cell Signaling Technology, cat. no. 8018), FoxO1 (Abcam, cat. no. ab179450), Gsk3b (Cell Signaling Technology, cat. no. 12456), p-Pik3r1 (Abcam, cat. no. ab182651), p-Akt (Cell Signaling Technology, cat. no. 9271), Nono (Abcam, cat. no. ab133574) and β-actin (Cell Signaling Technology, cat. no. 5125S) at 4 °C overnight. PVDF membranes were washed with TBST buffer three times, and then incubated with the secondary antibodies (Cell Signaling Technology, goat anti-mouse, cat. no. 96714; goat anti-rabbit, cat. no. 7074) with horseradish peroxidase (HRP) for 2 h at room temperature. The blots were washed again and detected with SuperSignal™ West Pico PLUS (Thermo Fisher Scientific, cat. no. 34577). The density of the band was calculated using ImageJ software. Because the blots were cut prior to antibodies hybridisation according to the corresponding strip position of protein marker. we could not provide the full-length blots image. We performed all the original and unprocessed blots in the Supplementary Information File, and marked the film edge with red dotted boxes.

### Integrated multi-omics analysis

The integrated multi-omics analysis included RNA-seq and ATAC-seq analysis. They were performed on the Illumina platform and aligned to the human genome (Rn6) using Bowtie2 program. The DEseq2 algorithm was applied to obtain differentially expressed genes (DEGs) with a false discovery rate (FDR, Benjamini & Hochberg correction) < 0.01, and |log fold-change (logFC)|> 1. Three biological replicates were used in RNA-seq experiments.

In ATAC-seq analysis, only unique mapped read was used for peak calling by MACS2 with q value < 0.05. The ChIPseeker was used for peak assignment annotation. Differentially accessible regions (DARs) calling was performed by DiffBind package with FDR < 0.05 and |Fold Change|> 1. Peaks were called for each sample using HOMER and individual peaks separated by < 200 bp were joined together. Motif analysis on peak regions was performed by HOMER function.

Gene Ontology (GO) enrichment analysis and KEGG pathways analysis of differentially expressed genes was performed with the “clusterProfiler” R package. Gene‑set enrichment analysis (GSEA) of the pre-ranking RNA-seq list based on log2-fold change was used to assess enrichment of the gene-sets.

The phastCons score, calculated by the multiple alignment algorithm (phastCons, version rn6 http://hgdownload.soe.ucsc.edu/goldenPath/rn6/phastCons20way/), was used to evaluate the conservation between H9c2 WT and Nono-KO cells.

### Statistical analyses

All experiments in this study were duplicated three times. Statistical analyses were performed using GraphPad software (GraphPad Prism 8.0.1), Microsoft Excel (Microsoft office 2013) and ImageJ software (ImageJ, 1.53c). The differences were analyzed via the Student's t-test welch's correction between Nono-KO and WT groups. Data were shown as Mean ± SD. ns, no significant. *, *P* < 0.05. **, *P* < 0.01.

## Supplementary Information

Supplementary Information 1.

Supplementary Information 2.

Supplementary Information 3.

Supplementary Information 4.

Supplementary Information 5.

Supplementary Information 6.

Supplementary Information 7.

Supplementary Information 8.

Supplementary Information 9.

## Data Availability

The detailed procedure and raw data of RNA-Seq and ATAC-seq analysis were presented in Supplementary dataset files and the NCBI Gene Expression Omnibus under accession code GSE211404 (Record GSE211404 remains in private status until May 01, 2023. The reviewer’s access number is ahypoiqcxfyhjyb).
