# Supplementary material for: Nono deficiency impedes the proliferation and adhesion of H9c2 cardiomyocytes through Pi3k/Akt signaling pathway
Source: Sci Rep. 2023 May 2;13:7134. doi: 10.1038/s41598-023-32572-x (PMC10154399; doi:10.1038/s41598-023-32572-x)
Supplement: Supplementary file 1 — Supplementary Information 1. [file 41598_2023_32572_MOESM1_ESM.docx]

Nono deficiency impedes the proliferation and adhesion of H9c2 cardiomyocytes through Pi3k/Akt signaling pathway

Yu-Qing Lei^1,2,3^, Zhou-Jie Ye^1, 2^, Ya-Lan Wei^1, 2^, Li-Ping Zhu^1, 2^, Xu-Dong Zhuang^1, 2^, Xin-Rui Wang^1, 2*^ and Hua Cao^1,2,3*^

^1^ Fujian Maternity and Child Health Hospital, College of Clinical Medicine for Obstetrics & Gynecology and Pediatrics, Fujian Medical University, Fuzhou 350000, China.

^2^ NHC Key Laboratory of Technical Evaluation of Fertility Regulation for Non-human Primate (Fujian Maternity and Child Health Hospital), Fuzhou 350000, China.

^3^ Department of Cardiac Surgery, Fujian Children's Hospital (Fujian Branch of Shanghai Children's Medical Center), College of Clinical Medicine for Obstetrics & Gynecology and Pediatrics, Fujian Medical University, Fuzhou 350011, China.

*** Correspondence author:** [wanxiru@sjtu.edu.cn](mailto:wanxiru@sjtu.edu.cn) (Xin-Rui Wang); [caohua69@fjmu.edu.cn](mailto:caohua69@fjmu.edu.cn) (Hua Cao)

Supplementary Figures 1 – 4

Supplementary Table 1

**
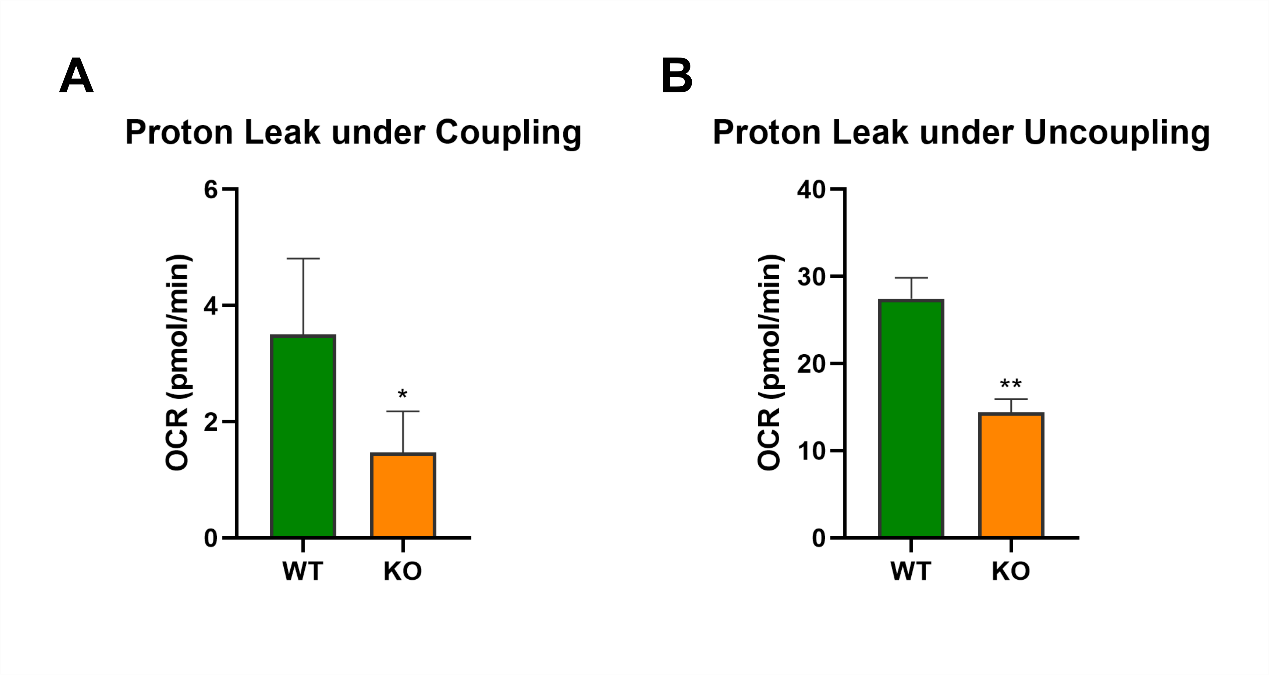
**

**Supplementary Figure 1. Nono gene deficiency in H9c2 cells blocked proton leak.** The proton leak was determined in two groups using the Seahorse equipment. The proton leak was calculated with OCR values under the coupling and uncoupling states. (a) Proton leak under the coupling state. (b) Proton leak under the uncoupling state. The mean values of three experiments were used in the bar figure for mean ± SD. *, p < 0.05; **, p < 0.01 vs. control.

**
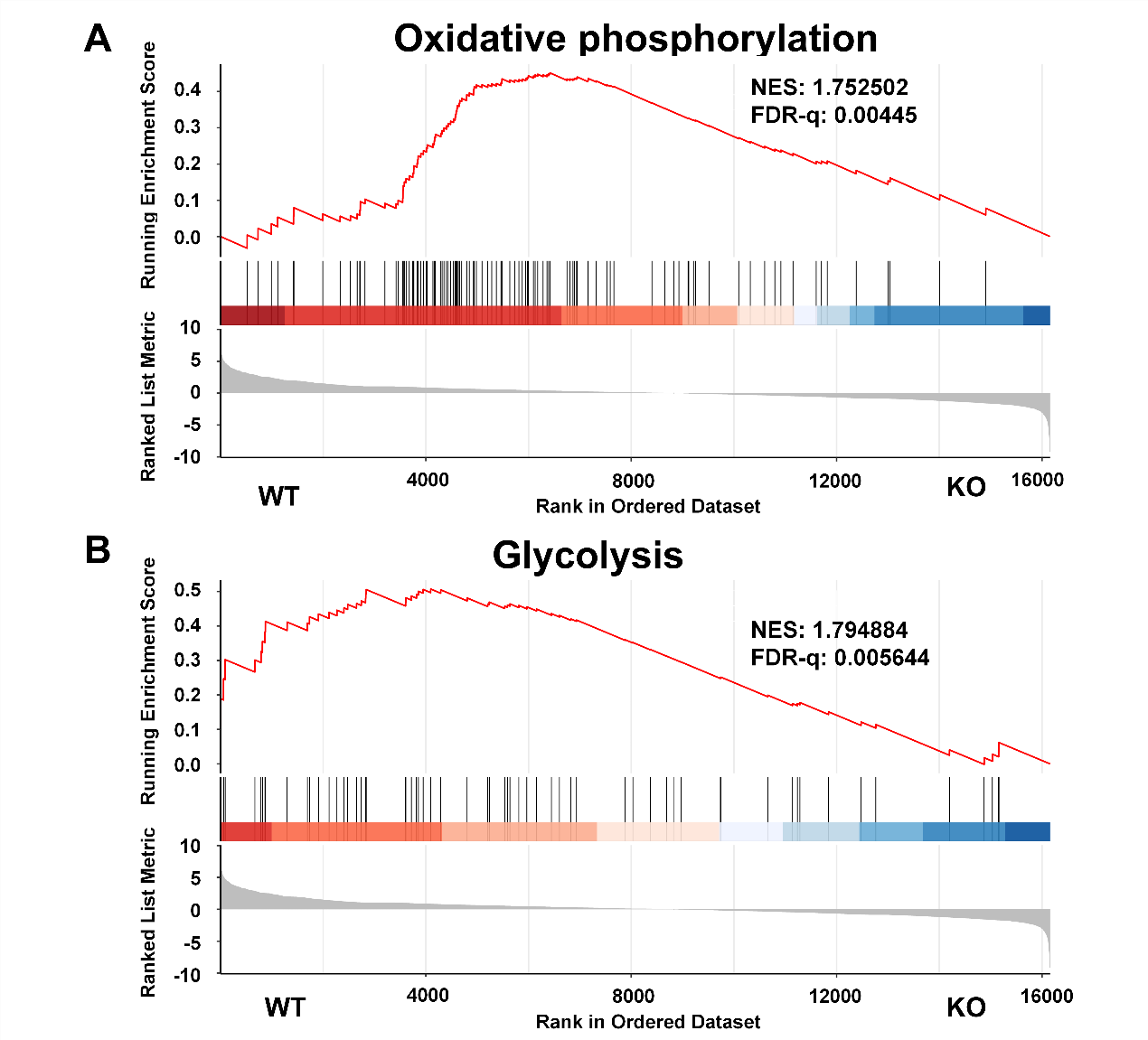
**

**Supplementary Figure 2. GSEA of RNA-seq analysis showing enrichment of the oxidative phosphorylation genes (a) and glycolysis genes (b).** NES, normalized enrichment score; FDR, false discovery rate**.**


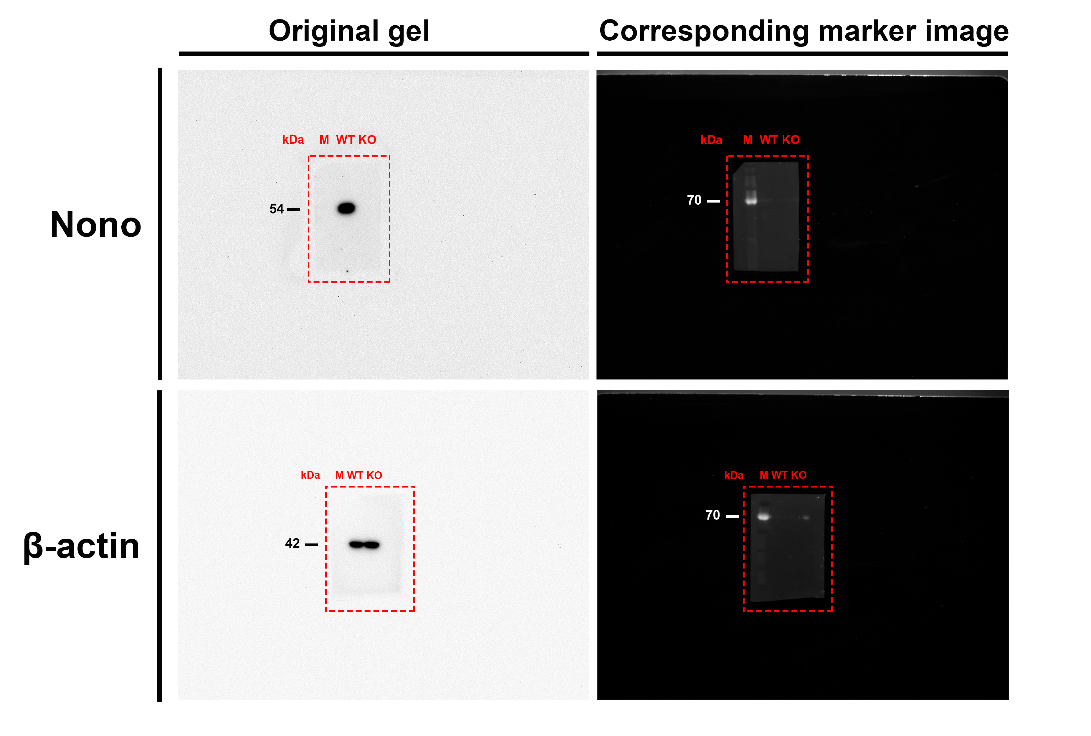


**Supplementary Figure 3. The original bands of Western blotting for Fig. 1d.**

**
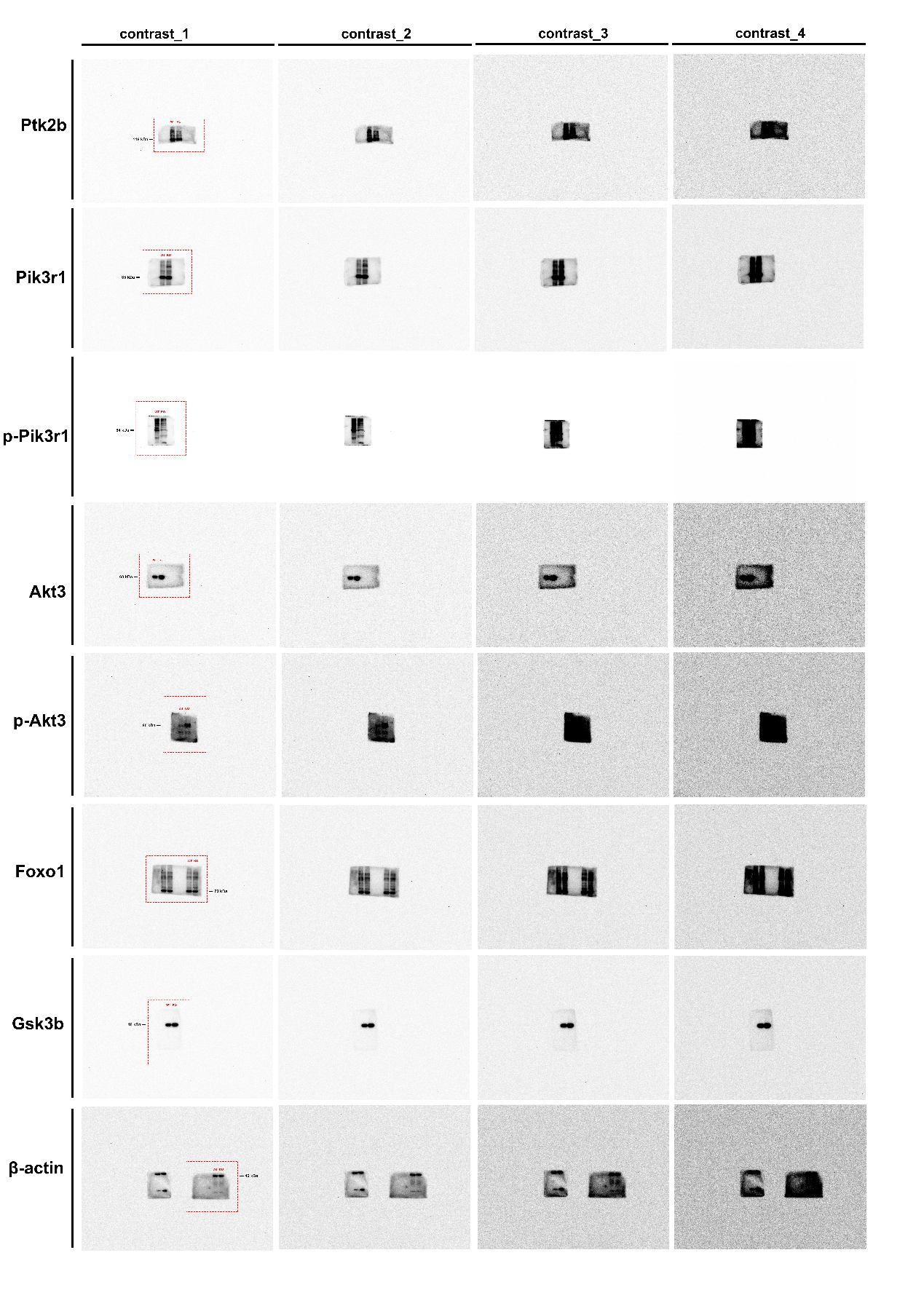
**

**Supplementary Figure 4. The original bands of Western blotting for Fig. 6b.**

**Supplementary Table 1. List of primers used for qPCR.**

| Genes | Primer sequence (5' to 3') | |
| --- | --- | --- |
| *Itga1* | Forward | AAGACCATAAGGGAGGCGTAT |
|  | Reverse | TCAGTCAGACCGTCACCATT |
| *Itga4* | Forward | GCAGCCTCGGTTACATATACG |
|  | Reverse | TCACTAAGGTCACTCTGTTGTCT |
| *Itga6* | Forward | GCCTCTCTCGTTCTACGCTC |
|  | Reverse | AACACCGTCACTCGAACCTG |
| *Itga8* | Forward | GCGTGCAAATCAACGTCACT |
|  | Reverse | CTGATGGTGCTGGGTCCAAT |
| *Itgav* | Forward | GAACAAGGAGAACCAGAACCATT |
|  | Reverse | TGACTAGCGTAGAGTTGAAGAGAT |
| *Itgb1* | Forward | ACAGATGAAGTGAACAGTGAAGAC |
|  | Reverse | GGACCTATCGCAGTTGAAGTTATC |
| *Cdk6* | Forward | TTCAGATGGCCCTTACCTCG |
|  | Reverse | GCAAAGATGCAGCCAACACT |
| *Cdkn1a* | Forward | TTGTCGCTGTCTTGCACTCT |
|  | Reverse | CTTGCAGAAGACCAATCGGC |
| *Ccnd3* | Forward | CTCCTACTTCCAGTGCGTGC |
|  | Reverse | AGGACAGGTAGCGATCCAGG |
| *Ccne1* | Forward | GTCAACGACACGGGAGAAGT |
|  | Reverse | AGCAGCGAGGACACCATAAG |
| *Ccnb1* | Forward | GCCTGAGCCTGAACCTGTTA |
|  | Reverse | TGGATCACCACCATCGTCTG |
| *Ccna2* | Forward | GGATGGTAGTTTTGAATCACC |
|  | Reverse | TTAGTGATGTCTGGCTGCCTC |
